# Cytochrome P450 *CYP6EV11* in *Chironomus kiiensis* Larvae Involved in Phenol Stress

**DOI:** 10.3390/ijms19041119

**Published:** 2018-04-09

**Authors:** Qihui Zhang, Dong Chu, Lili Sun, Chuanwang Cao

**Affiliations:** College of Forestry, Northeast Forestry University, Harbin 150040, China; secertzhang@163.com (Q.Z.); dongtianderuguo@163.com (D.C.); sunlilinefu@126.com (L.S.)

**Keywords:** *CYP6EV11*, phenol stress, *Chironomus kiiensis*, molecular biomarker

## Abstract

Phenol is one of the organic pollutants which can cause water environment pollution. It is not only enriched in aquatic organisms but is also a serious threat to human health. *Chironomus kiiensis* is very sensitive to the contaminants in water and its cytochrome P450s are usually chosen as biomarkers for water pollution. To examine whether *CYP6EV11* plays a role in the oxidative metabolism of phenol, we measured the silencing efficiency of *CYP6EV11* and evaluated larval susceptibility to sublethal phenol levels by RNA interference (RNAi) technology. The results showed that the transcription of *CYP6EV11* was found significantly up-regulated when the 4th instar *C.* *kiiensis* larvae were exposed to three doses of phenol. However, the transcriptional levels of *CYP6EV11* were significantly suppressed by 92.7% in the 4th instar *C. kiiensis* larvae soaked in *dsCYP6EV11* compared with those soaked in *dsGFP* for 6 h. The *CYP6EV11* expression and mortality of the 4th instar *C. kiiensis* larvae with *CYP6EV11* silencing were mostly decreased under phenol stress. Therefore, the *CYP6EV11* gene may be used as a molecular biomarker for earlier warning and monitoring for water pollution.

## 1. Introduction

Phenol (carbolic acid, phenolic acid, phenylic acid or oxybenzene) consists of an aromatic ring linked a hydroxyl group and is widely used as the precursor to produce industrial compounds such as kerosene, phenolic resin and pesticides [1]. The compounds penetrat ecosystems due to the drainage off municipal or industrial sewage to surface water [2]. The Environmental Protection Agency (EPA) specified the standard maximum of phenol contaminant level of 1 mg/L in wastewater [3]. Since the phenolic compounds were stable over a long term and harmful to organisms at low dose, many of them have been classified as hazardous pollutants due to their harmful damage to human health [4]. Although the standards of critical toxic concentration of phenol were much different, and varied from 1.06 μM to 105.24 μM (105.24 mM in Malaysia,10.63 mM in the United States, 1.06 mM in Australia) [5,6,7], there is no doubt that phenol is harmful and should be monitored. Our previous research also found that the enzymatic activities in *Propsilocerus akamusi* were significantly altered in response to phenol [8]. 

Cytochrome P450s (CYPs) belong to a superfamily, which is found in all organisms that play an important role in many physiological processes such as the metabolism of fatty acids, steroids and vitamin D as well as some other phytochemicals like pesticides [9]. It is well documented that CYPs are associated with the process of detoxification in insects, and the elevated activity of CYP enzymes has the ability to accelerate metabolism of pesticides. The CYP6 families in terrestrial invertebrates have frequently been shown to play a role in the detoxification of xenobiotics and metabolic resistance to insecticides [10,11,12,13,14,15]. Most CYP6 genes in insects have been shown to enhance metabolic detoxification, such as *CYP6AB37*, *CYP6AB35*, *CYP6B53*, *CYP6AB3*, *CYP6AB32*, *CYP6AB33*, *CYP6AB36*, *CYP6CT4* and *CYP6AN15v1* of *Lymantria dispar* [15]. It has been reported that *CYP6BQ23* in *Meligethes aeneus* is associated with the resistance to deltamethrin [16]. In several species of Chironomus, different toxic compounds are affected by the expression of the *CYP6* gene. For example, the mRNA levels of *CYP6B7* in *Chironomus riparius* are evaluated after exposures to the ultraviolet filters benzophenone-3 (BP3) and 4-methylbenzylidene camphor (4MBC) [17] and the antibacterial agent triclosan [18]; in *C. tentan*s the transcriptional activities of *CYP6EX3* and *CYP6EV1*, *CYP6EV3* have been studied after atrazine and chlorpyrifos exposures. Moreover, the present study has reported the effects of phenol on the expression of ten *CYP6* genes in *C. kiinensis* [19]. On the other hand, other cytochromes in different Chironomus species have been analyzed as possible biomarker genes that could be useful in ecotoxicological studies, risk assessment and bioremediation, such as *CYP4G* [20], *CYP9AT2* [21], *CYP4D2*, *CYP9F2* and *CYP12A2* in *C. riparius* [18]; *CYP4DG1*, *CYP4DG2* and *CYP9AT1* in *C. tentans* [22,23].

Chironomidae are known as non-biting midges belonging to a family of Diptera: Nematocera. They often distribute in urban and residential areas in close proximity to polluted and eutrophic waters causing a big problem worldwide [24]. *Chironomus kiinensis* is broadly distributed in Malaysia, Japan, USA and South China [25]. *C. kiinensis* could be used extensively for acute or chronic bioassays in fresh water ecosystems as it has a relatively short life cycle, and due to the ease of maintenance of laboratory cultures and relative sensitivity to aquatic contaminants [26]. To provide molecular evidence of CYP gene detoxification that will be of benefit to further monitor water pollution, we: (1) examined the transcriptional responses of *CYP6EV11* in *C. kiiensis* to the exposure of phenol at different concentration; (2) revealed the phenol-induced down-regulation of *CYP6EV11* contributing to decreased toxicity of phenol to *C. kiiensis* using the RNA interference (RNAi) method. These results may potentially develop sensitive molecular markers of Chironomidae for monitoring pesticide exposures in non-target organisms in aquatic systems.

## 2. Results

### 2.1. cDNA Cloning and Characterization

In databases, full-length cDNA of *CYP6EV11* was detected in the P450 family genes, with open reading frames (ORFs) of 1476 bp encoding 491 amino acids, with predicted molecular masses of 56.79 kDa and isoelectric points (PI) of 9.12. Besides, there are no signal peptides in *CYP6EV11*.

### 2.2. Polygenetic Analysis

**Figure 1:**
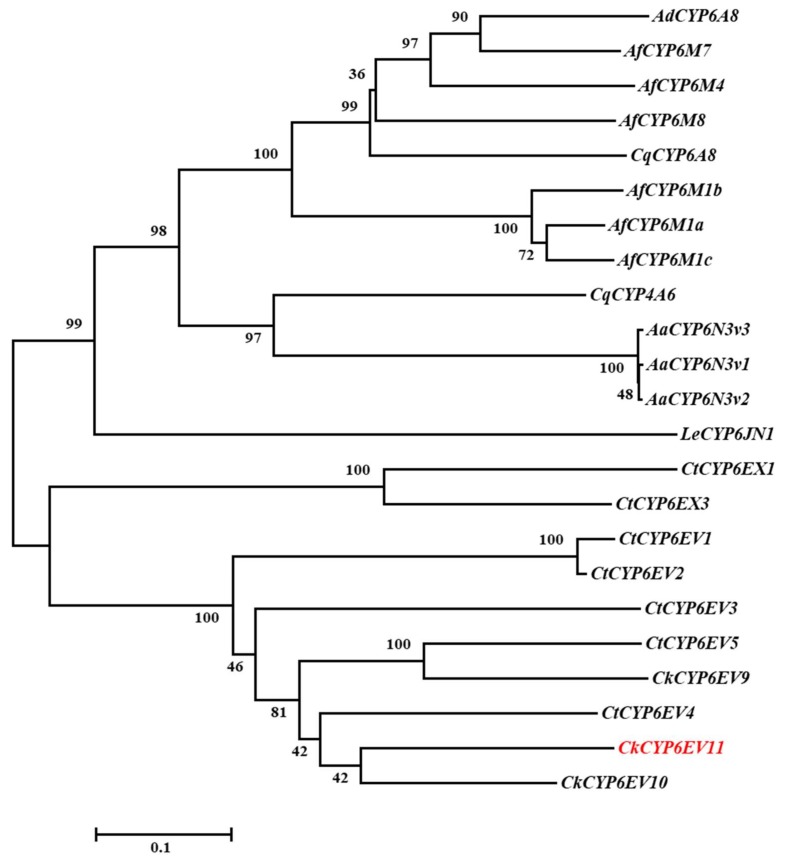
Phylogenetic tree of 23 CYP genes are from 7 insects. These genes were downloaded from the National Center for Biotechnology Information (NCBI) databases. The gene accession numbers are parenthesized. The CYP genes are *CtCYP6EV1* (ARO50426.1), *CtCYP6EV2* (ARO50430.1), *CtCYP6EV3* (ARO50428.1), *CtCYP6EV4* (ARO50429.1), *CtCYP6EV5* (ARO50442.1), *CtCYP6EX1* (ARO50434.1), *CtCYP6EX3* (ARO50425.1), *CkCYP6EV9* (AHJ10930.1), *CkCYP6EV10* (AHJ10931.1), *CqCYP4A6* (XP_001867280.1), *CqCYP6A8* (XP_001870174.1), *AaCYP6N3v1* (AAF97936.1), *AaCYP6N3v2* (AAF97937.1), *AaCYP6N3v3* (AAF97938.1), *AdCYP6A8* (ETN65670.1), *AfCYP6M1a* (AFM08393.1), *AfCYP6M1b* (AFM08394.1), *AfCYP6M1c* (AFM08395.1), *AfCYP6M4* (AFM08397.1), *AfCYP6M7* (AIE17403.1), *AfCYP6M8* (AFM08398.1), *LeCYP6JN1* (ALX81394.1), *CkCYPEV11*. *CkCYP6EV11* is a target gene in this study from *Chironomus kiiensis,* which was obtained from transcriptome sequencing in our previous study [26]. These genes are from *Chironomus tentans, Chironomus kiiensis*, *Culex quinquefasciatus*, *Aedes albopictus*, *Anopheles darling*, *Anopheles funestus* and *Liposcelis entomophila.*

**Figure 2:**
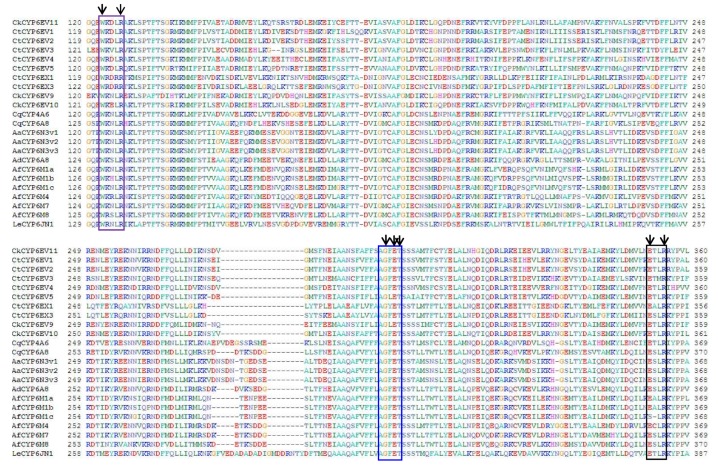

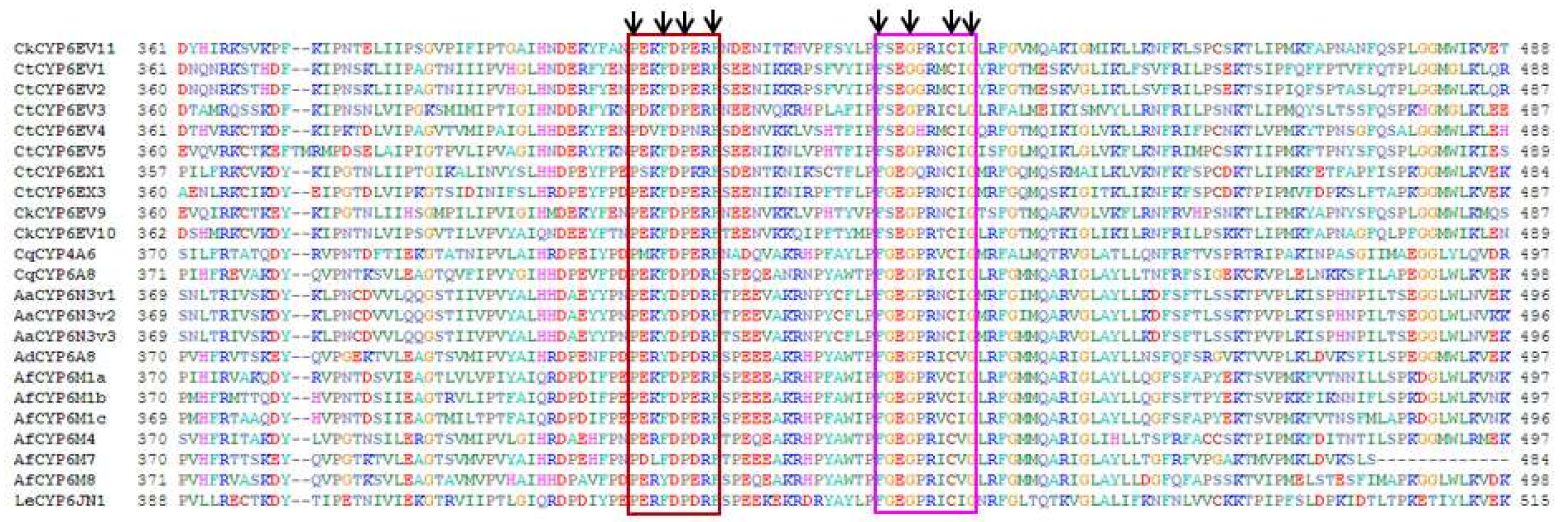
The alignment of deduced amino acid sequences from 23 CYP genes from seven insects. The five important conserved motifs of the CYPs were framed by only partial sequence alignment. The purple box shows helix-C with conserved sequences WxxxR; blue box indicates helix-I with typical sequences AGxET; and dark box highlights helix-K with sequences E/SxLR. The heme-binding domain is boxed in pink with typical residues FxxGxxxCxG/A and the conserved Meander domain was boxed in red with conserved sequences PxxFxPxxF. The similarity of *CYP6EV11* with *CkCYP6EV10*, *CtCYP6EV4*, *CtCYP6EV5*, *CkCYP6EV9*, *CtCYP6EV2*, *CtCYP6EV3*, *CtCYP6EV1*, *CtCYP6EX3*, *AfCYP6M8*, *CqCYP6A8*, *AfCYP6M4*, *AdCYP6A8*, *AaCYP6N3v3*, *AaCYP6N3v2*, *AaCYP6N3v1*, *AfCYP6M7*, *CqCYP4A6*, *AfCYP6M1b*, *AfCYP6M1a, CtCYP6EX1*, *LeCYP6JN1*, *AfCYP6M1c* is 71%, 64%, 63%, 61%, 58%, 56%, 56%, 47%, 43%, 44%, 44%, 43%, 42%, 41%, 42%, 43%, 42%, 45%, 45%, 44%, 41%, 44%, respectively.

Based on the identities of *CYP6EV11*, phylogenetic trees were constructed with 22 genes of high homologous amino acids in insects. The *CYP6EV11* and *CYP6EV10* (AHJ10931.1) in *C. kiiensis* shared the highest sequence similarity (71%), and were clustered into a group. All the 22 CYP sequences have been deposited in the NCBI database with their accession numbers as shown in the Figure 1. Five similar motifs were found in typical CYPs, including helix-C, helix-I, helix-K, Meander domain and heme-binding domain from N to C terminal (Figure 2). Helix-C is heme-interacting region with typical sequences WxxxR; AGxET motif is located in helix-I and reportedly to make an oxygen binding pocket; E/SxLR located in helix-K with the hydrogen bonding domain and PxxFxPxxF motif are thought to form a set of salt bridge interactons (E-R-R) for stabilizing the structure of protein [27]; and the P450 heme-binding domain locates at the 3′-end with the FxxGxRxCxG/A sequences [28,29].

### 2.3. Expression Profiling under Phenol Stress

**Figure 3:**
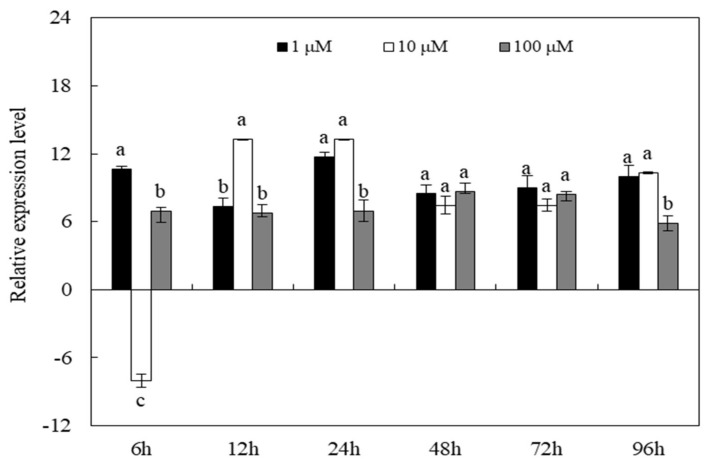
Transcriptional profiles of *CYP6EV11* in the 4th instar *C. kiiensis* larvae following exposure to three doses of phenol (1, 10 and 100 µM) during a 96-h period. The larvae without phenol treatment were regarded as controls. The standard error (SE) bars were calculated based on three experimental replicates. The bars with different letters (a–c) are significantly different at *p* < 0.05 based on one-way ANOVA followed by Duncan multiple comparisons. All of the relative expression levels were log2 transformed.

QRT-PCR analysis was performed to compare the transcription levels of *CYP6EV11* under the three doses of phenol stresses. The *CYP6EV11* was significantly up-regulated by 1 and 100 µM of phenol, respectively. However, *CYP6EV11* was suppressed by phenol at the dose of 10 µM at 6 h. After the larvae were stressed for 12~96 h, the expressions of *CYP6EV11* were significantly up-regulated under three doses of phenol stresses. As the results of induced expression of *CYP6EV11* at 1 µM phenol stress, 1 µM was chosen as the dose of treatment to explore the effects of gene silencing on development and response to phenol stress (Figure 3).

### 2.4. Gene Silencing Analysis

**Figure 4:**
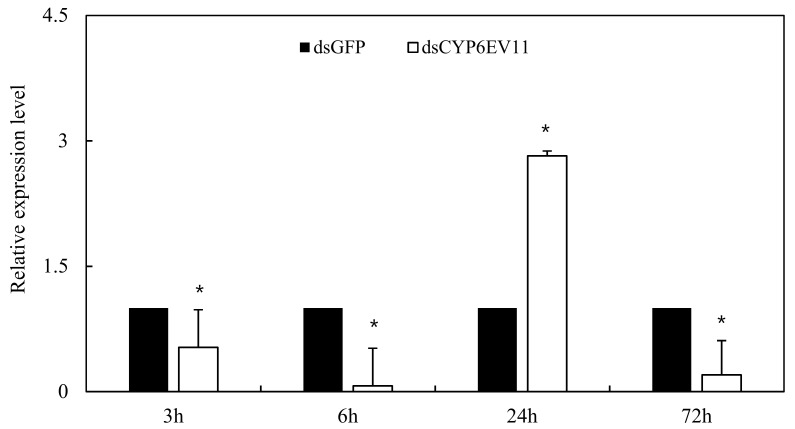
The *CYPEV11* transcriptional level in *C. kiiensis* soaked in *dsCYPEV11* compared with those soaked in *dsGFP*. The asterisk (*) on the SE bars indicate significant differences between treatments and controls (*p* < 0.05).

To determine whether *dsCYP6EV11* could inhibit the expression of *CYP6EV11*, the 4th instar larvae of *C. kiiensis* were soaked in *dsCYP6EV11* and larvae soaked in *dsGFP* were chosen as the controls; qRT-PCR analyses showed that the *CYPEV11* transcript level was reduced at three of four times, especially, the *CYPEV11* transcript level was reduced by 92.7% at 6 h compared with those soaked in *dsGFP* (*p* < 0.05). However, the *CYPEV11* transcript level was increased by 182.3% at the time point of 24 h (Figure 4). 

**Figure 5:**
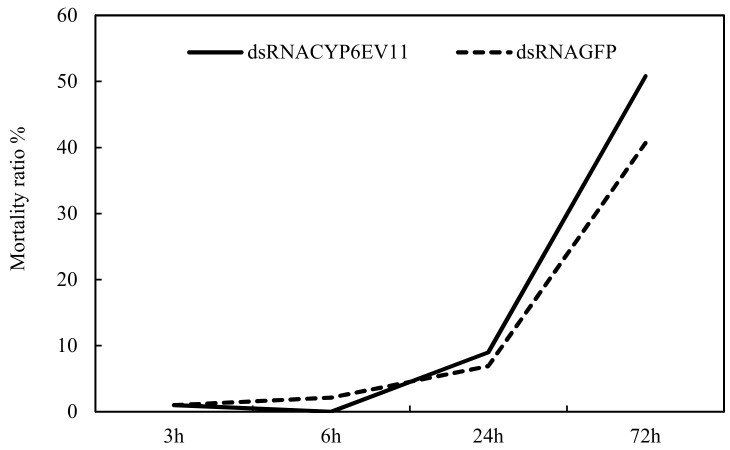
The mortality rate of *C. kiiensis* larvae with *CYP6EV11* gene silencing and the groups soaked in *dsGFP*.

Meanwhile, the mortality rate was recorded to explore the effects of gene silencing on the growth of larvae. The results showed that the mortality rate of each treatment gradually increased with the increasing of silencing time. However, there were no statistical differences in the mortality rates between the larvae soaked in *dsCYPEV11* and *dsGFP* at the period of 3~24 h. The mortality rate of larvae soaked in *dsCYP6EV11* reached the highest at 72 h, which was 24.9% more than observed for the *dsGFP* treatment (Figure 5). 

### 2.5. Effects of Gene Silencing on Development and in Response to Phenol Stress

**Figure 6:**
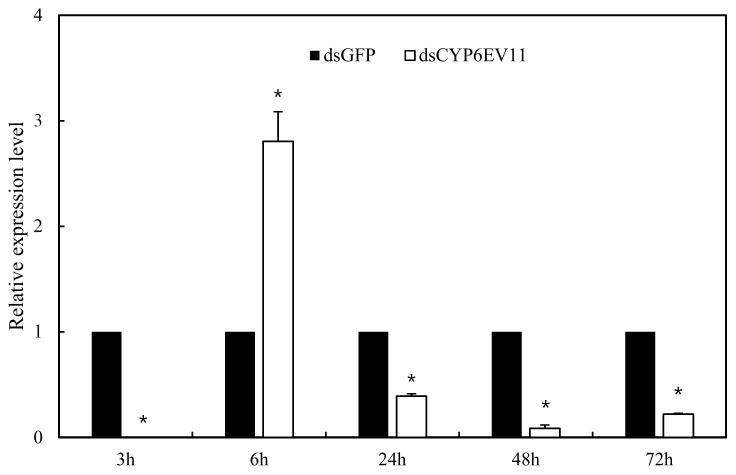
Transcriptional levels of *CYP6EV11* in silenced *C. kiiensis* larvae in response to phenol stress. The larvae treated with *dsGFP* were regarded as control. The SE bars were calculated based on three experimental replicates. The asterisk (*) on the SE bars indicate significant differences between treatments and controls (*p* < 0.05).

Since the transcript levels of *CYP6EV11* were successfully suppressed in larvae soaked in *dsRNA*, we further examined whether the suppression of *CYP6EV11* transcript had effects on *CYP6EV11* in response to phenol stress. *CYP6EV11* was mostly suppressed after 3-h treatment, the transcript level was reduced by 99.9% compared with what was observed in those soaked in *dsGFP* (*p* < 0.05). However, the *CYPEV11* transcript level was increased by 180.5% compared with the larvae soaked in *dsRNA* for 6 h. After 72 h of stress, the susceptibility of larvae to phenol gradually decreased and the transcript level was reduced by 77.9% (Figure 6).

**Figure 7:**
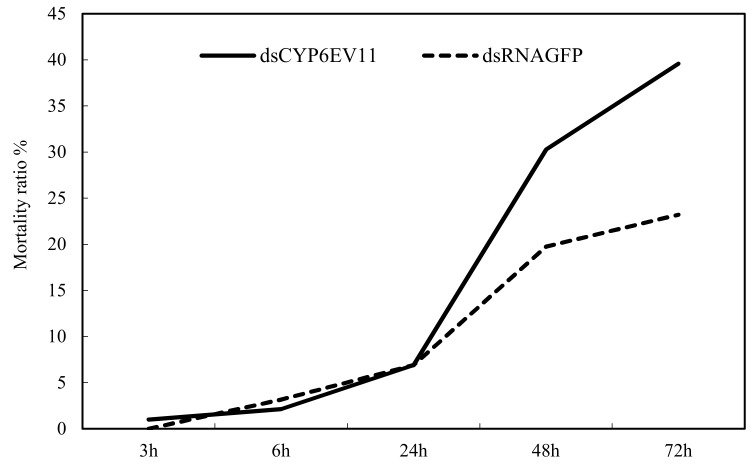
The mortality rates of *C. kiiensis* larvae with *CYP6EV11* gene silencing under phenol stress. The larvae were soaked in the mixture phenol at 1 µM and *dsRNA* at 2 µg/µL, and the groups soaked in *dsGFP* were regarded as controls to compare the susceptibility to phenol after *CYP6EV11* gene silencing.

The mortality rates of *dsCYP6EV11* and *dsGFP* treatments gradually increased with the increase of silencing time. After the larvae were treated with a mixture (phenol at 1 µM and *dsRNA* at 2 µg/µL) for 24 h, all the mortalities of *dsCYP6EV11* groups were significantly higher than those of *dsGFP* groups (*p* < 0.05, Figure 7). Especially, the mortality of *dsCYPEV11* treatment increased by 70.5% compared with the larvae soaked in *dsGFP* at the 72-h time point.

## 3. Discussion

Insect cytochrome P450s are known to play an important role in detoxifying insecticides and plant toxins [15,30]. The up-regulation of CYPs, especially the members of the CYP6 family, has been confirmed to be associated with enhanced metabolic detoxification of insecticides in insects [16,31,32,33,34]. In this study, the transcripts of *CYP6EV11* treated with phenol were found to be significantly up-regulated compared to those in the untreated groups. The increased expression of *CYP6EV11* may imply an enhanced ability to metabolize exogenous compounds. Our pervious study also found that the *CYP6FV2* and *CYP6FV1* in 4th instar *C. kiinensis* larvae were mainly up-regulated during a 96-h phenol exposure [19]. As a result of stress, similar differential transcriptional expression levels have been reported in *C. tentans*. Tang et al. (2018) reported stress-related genes in *C. tentans*, including two cytochrome P450 genes (*CYP6EV1* and *CYP4DG2*), have considerable potential as sensitive biomarkers for the diagnosis of chlorpyrifos contamination [22]. The expressions of *CYP6EX3* and *CYP6EV3* in *C. tentans* can also be significantly up-regulated by atrazine at 1000 and 5000 mg/L, respectively [23]. Thus, upregulation of the *CYP4G* gene in *Chironomus riparius* was found after exposures to TBTO (1 ng/L 24 h–0.1 ng/L 96 h) [20]. 

To reveal the role of *CYP6EV11* in pollutant metabolism, RNAi technology was used in this study. RNAi has been successfully used in most insects like Lepidoptera and Coleoptera, but rarely used in Chironomidae [35,36,37]. Lu et al. (2012) had successfully revealed two acetylcholinesterase genes (*TcAce1* and *TcAce2*) in *Tribolium castaneum* by the method of gene silencing [38]. Knockdown of *CYP6EV11* was successfully conducted by this method, and a similar method was also applied for *CYP6EX3* silencing to investigate the susceptibility of *C. tentans* larvae to chlorpyrifos [23]. Compared with dsGFP groups, *CYP6EV11* expression was significantly decreased in *C. kiiensis* larvae soaked in *dsCYP6EV11* under phenol and non-phenol stresses. The results showed that *CYP6EV11* played a role in oxidative metabolism to phenol. Interestingly, the *C. kiiensis* larvae decreased susceptibility to phenol when the *CYP6EV11* was silenced resulted in low mortality indicating other *CYP* family genes may be triggered to increase resistance to phenol stress. This result is consistent with the report of Cao et al. (2016) [19]. RNAi technology with *dsRNA* soaking was used to further study gene function of Chironomidae. Our results have provided, for the first time, crucial evidence with regard to which *CYP6EV11* in *C. kiiensis* may be a new molecular biomarker for monitoring phenol pollution and, therefore, the extension in other species is available. Further studies should validate the metabolism ability by heterologously expressed *CYP6EV11* in *C. kiiensis*.

## 4. Materials and Methods 

### 4.1. Experimental Midge Rearing

The *Chironomus kiiensis* were obtained from Shenzhen Municipal Water Affairs Bureau, China, and were cultured according to the method of Cao et al. (2013) [26]. Briefly, the *C. kiiensis* were reared in mixed-age cultures by generation to generation under the condition of 20 ± 2 °C and L16:D8. The midges were fed with goldfish granules (Beijing San You Beautification Free TECH. Co., Ltd., Beijing, China) and maintained in a glass tank (50 cm × 20 cm × 30 cm) that was covered with nylon net. 

### 4.2. Cloning and Identification of CYP6EV11

Total RNA was isolated using an RNeasy Mini Kit (Qiagen, Valencia, CA, USA) following the manufacturer′s guidelines and treated with RNase-free DNase I (Qiagen, Madison, WI, USA). RNA concentrations were measured using a spectrophotometer, and RNA integrity was checked by analysis on a 1.0% *w*/*v* agarose gel. The *C. kiinensis* transcriptome was profiled by conducting Solexa sequencing at the Beijing Genomics Institute (BGI) (Shenzhen, China) [26].

The cDNA of *CYP6EV11* was cloned by the method of RACE using 3′-Full RACE Kit and 5′-Full RACE Kit (TaKaRa, Kyoto, Japan), and was purified using E.Z.N.A. Gel Extraction Kit (Omega, Norcross, GA, USA). After purity and quality checks, the open reading frames (ORFs) were confirmed using the ORF finder (Available online: http://www.ncbi.nlm.nih.gov/gorf.html). The molecular masses, isoelectric points (PI) and the conserved domains were derived using ProtParam (Available online: http://au.expasy.org/tools/protparam.html) and Conserved Domains (Available online: http://www.ncbi.nlm.nih.gov/Structure/cdd/wrpsb.cgi) of NCBI, respectively. SignalP3.0 Server (Available online: http://www.cbs.dtu.dk/services/SignalP) was used to compute signal peptide of the CYP6EV11 h.

### 4.3. Multiple Sequence Alignment and Polygenetic Analysis

Amino acid sequences corresponding to CYP6 in other insects were retrieved from the NCBI database (Available online: http://www.ncbi.nlm.nih.gov/BLAST/) for multiple sequence alignment using CLUSTALX 1.83(Institut de Genetique et de Biologie Moleculaire et Cellulaire, Illkrich, France). The phylogenetic tree was constructed by the neighbor-joining method and bootstrapped with 1000 replicates to evaluate the branch strength using MEGA 5.1 software(CEMI, Temp, AZ, USA) [39].

### 4.4. C. kiiensis Larvae Stress and RNAi Analysis

The larvae were exposure to phenol (0, 1, 10, 100 μM) with ten replicates of each treatment. Thirty 4th instar larvae with similar size and body color were randomly assigned in each replicate. The controls were maintained without any exposure to chemicals for the different durations and concentrations along with the phenol-exposed samples. After exposure, two living larvae were randomly collected from each replicate at 6, 12, 24, 48, 72 and 96 h, and immediately frozen in liquid nitrogen before being stored at −80 °C Twenty frozen midges were randomly selected from each treatment at each time interval for RNA preparations. 

The *dsRNAs* were synthesized with cDNA of *CYP6EV11* and *GFP* using MEGAscript T7 Kit (Ambion, Austin, TX, USA) following the manufacturer′s instructions and were purified with ammonium acetate, water saturated phenol and chloroform. The *dsRNA* was resuspended in RNase-free water, and quantitated at 260 nm using Nanodrop 2000 Spectrophotometer (Thermo Fisher Scientific Inc., Carlsbad, CA, USA). The quality and integrity of *dsRNA* were verified by 1.0% agarose gel electrophoresis. The larvae were soaked in *dsRNA* of *CYP6EV11* (2 µg/µL), ten replicates containing thirty 4th instar larvae in each replicate. The controls were soaked in *dsRNA* of *GFP* (2 µg/µL) along with the *dsRNA* of *CYP6EV11* samples. Two living larvae were collected for each replicate at 3, 6, 24 and 72 h, respectively, and then stored at −80 °C for RNA extraction to measure the silence efficacy. To explore *CYP6EV11* silencing in response to phenol stress, the survival larvae after soaking with the 1µM phenol for 1 h were soaked in the mixture of 2 µg/µL *CYP6EV11 dsRNA* and 1 µM phenol. Twenty healthy larvae were collected at 3, 6, 24, 48, and 72 h, respectively, and stored at −80 °C for RNA extraction to obtain *CYP6EV11* gene expression profiles. The groups soaked in the mixture of 2 µg/µL *GFP dsRNA* and 1 µM phenol were regarded as controls. All mortalities were recorded among treatments.

### 4.5. Real-time RT-PCR Analysis

Approximately 1 μg of total RNA was reverse transcribed to cDNA using 1 μM oligodeoxythymidine primer. Synthesized cDNAs were diluted to 100 μL with sterile water and used as template for real-time PCR. The following primers were designed for amplification of the *CYP6EV11* gene, F: 5′-GGCGGACAAGAATGGAAAGA-3′ and R: 5′ -GGCTGTCCAAGACACTTGAT-3′. The Actin (F: 5′-AATGGGATCGCTTGGGTGCTTT-3′ and R: 5′-TCAGCTTCACCCAATGTTGCCT-3′) was selected as internal controls to calculate the relative expression level by the method of delta–delta CT method [40] and 2^−ΔΔ*C*t^ [41]. Amplifications were performed with the following parameters: 94 °C for 30 s followed by 45 cycles at 94 °C for 12 s, 60 °C for 30 s, 72 °C for 40 s, and 82 °C for 1 s for plate reading.

## 5. Conclusions

The *CYP6EV11* in *C. kiiensis* was firstly identified and was found to be mostly upregulated under phenol stress. Compared with *dsGFP*, the *CYPEV11* was effectively 92.7% silenced by RNAi in 4th instar *C. kiiensis* larvae soaked in *dsCYP6EV11* for 6 h. The *CYP6EV11* transcript level and susceptibility of the *C. kiiensis* larvae were markedly decreased under phenol stress after *CYP6EV11* silencing. Therefore, the *CYP6EV11* gene may be used as a sensitive molecular marker for phenol pollution monitoring.
